# Effects of Exergaming on Morphological Variables, Biochemical Parameters, and Blood Pressure in Children and Adolescents with Overweight/Obesity: A Systematic Review with Meta-Analysis of Randomized Controlled Trials

**DOI:** 10.3390/children12010029

**Published:** 2024-12-27

**Authors:** Jordan Hernandez-Martinez, Joaquín Perez-Carcamo, Hassan Melki, Izham Cid-Calfucura, Edgar Vasquez-Carrasco, Pedro Delgado-Floody, Claudio Romero, Tomás Herrera-Valenzuela, Braulio Henrique Magnani Branco, Pablo Valdés-Badilla

**Affiliations:** 1 Department of Physical Activity Sciences, Universidad de Los Lagos, Osorno 5290000, Chile; jordan.hernandez@ulagos.cl (J.H.-M.); joaquinalejandro.perez@alumnos.ulagos.cl (J.P.-C.); claudio.romero@ulagos.cl (C.R.); 2 Programa de Investigación en Deporte, Sociedad y Buen Vivir, Universidad de los Lagos, Osorno 5290000, Chile; 3 G-IDyAF Research Group, Department of Physical Activity Sciences, Universidad de Los Lagos, Osorno 5290000, Chile; 4 Higher Institute of Sport and Physical Education of Ksar Saïd, Manouba 2037, Tunisia; hmelki@yahoo.fr; 5 Department of Physical Activity, Sports and Health Sciences, Faculty of Medical Sciences, Universidad de Santiago de Chile (USACH), Santiago 8370003, Chile; izham.cid@gmail.com (I.C.-C.); tomas.herrera@usach.cl (T.H.-V.); 6 School of Occupational Therapy, Faculty of Psychology, Universidad de Talca, Talca 3465548, Chile; edgar.vasquez@utalca.cl; 7 Centro de Investigación en Ciencias Cognitivas, Faculty of Psychology, Universidad de Talca, Talca 3465548, Chile; 8 Department of Physical Education, Sport, and Recreation, Universidad de La Frontera, Temuco 4811230, Chile; pedro.delgado@ufrontera.cl; 9 Graduate Program in Health Promotion, Cesumar University (UniCesumar), Maringá 87050-900, Brazil; braulio.branco@unicesumar.edu.br; 10 Department of Physical Activity Sciences, Faculty of Education Sciences, Universidad Católica del Maule, Talca 3530000, Chile; 11 Sports Coach Career, School of Education, Universidad Viña del Mar, Viña del Mar 2520000, Chile

**Keywords:** virtual reality exposure therapy, exercise, child, pediatric obesity, biomarkers

## Abstract

Objectives: This systematic review with meta-analysis aimed to evaluate the available body of published peer-reviewed studies on the effects of exergaming (EXG) compared to the control group (CG) on morphological variables, biochemical parameters, and blood pressure in children and adolescents with overweight/obesity. Methods: A systematic literature search was conducted until September 2024 using five databases: PubMed, Medline, CINAHL Complete, Scopus, and Web of Science. PRISMA, TESTEX, RoB 2, and GRADE tools assessed the methodological quality and certainty of evidence. Hedge’s g effect sizes (ES) for morphological, biochemical, and blood pressure variables were calculated for meta-analyses. Using a random effects model, potential sources of heterogeneity were selected, including subgroup analyses (age) and single training factor analysis (program duration, training frequency). The protocol was registered in PROSPERO (code: CRD42024626992). Results: Out of 72 records, 6 randomized controlled trials with 191 children and adolescents with overweight/obesity were included. Nine meta-analyses were performed, showing significant decreases in body mass index (*p* = 0.04), waist circumference (*p* = 0.03), and systolic blood pressure (*p* = 0.007). However, no significant improvements were observed in diastolic blood pressure, body fat percentage, total cholesterol, HDL-cholesterol, LDL-cholesterol, triglycerides, and glucose. Subgroup analyses showed significant decreases in total cholesterol (<15 years, ES = 0.56; *p* = 0.006), HDL-cholesterol (<15 years, ES = 0.51; *p* = 0.01), LDL-cholesterol (<15 years, ES = 0.63; *p* = 0.01), and triglycerides (<15 years, ES = 0.82; *p* = 0.000). In training duration, only significant decreases in total cholesterol (ES = 0.69; *p* = 0.02) were presented in favor of <12 weeks vs. ≥12 weeks. While in training frequency only significant decreases in triglycerides (ES = 0.70; *p* = 0.03) were reported in favor of ≥3 sessions per week vs. <3 sessions per week. Conclusions: EXG significantly decreases body mass index, waist circumference, and systolic blood pressure in children and adolescents with overweight/obesity.

## 1. Introduction

Children and adolescent obesity have increased worldwide, with 158 million obese people and an expected rise to 254 million by 2030 [1]. Currently, the global economic impact of childhood obesity is estimated at $23.75 billion, with projections of increasing to $49.02 billion by 2050 [2]. Overweight and obesity are now considered a modifiable pandemic [3], associated with physical inactivity and risk factors such as hypertension, hyperglycemia, dyslipidemia, hypertriglyceridemia, and metabolic syndrome in children and adolescents [4,5], increasing the risk of stroke and early mortality in children and adolescents with obesity [6]. Therefore, it is vital to carry out interventions that increase physical activity practice in this age group to reduce these risk factors associated with overweight and obesity [7].

Interventions using resistance training, endurance training, high-intensity interval training (HIIT), or a combination of these interventions increase physical activity levels and decrease risk factors in overweight/obese children and adolescents according to García-Hermoso et al. [8] in a network meta-analysis. In a meta-analysis conducted by Liu et al. [9] in children and adolescents with overweight/obesity, significant decreases in body mass index (BMI, *p* < 0.01), waist circumference (*p* < 0.01), and body fat percentage (*p* < 0.01) were found in favor of resistance training interventions combined with aerobic training compared to inactive control groups (CG). Results similar to those presented in a meta-analysis conducted by Zhu et al. [10] in school children with obesity and cardiometabolic risk factors showed significant decreases in triglycerides, LDL cholesterol (*p* < 0.0001), and systolic blood pressure (SBP, *p* = 0.02) in favor of HIIT interventions compared to control groups. Interventions using aerobic, resistance training, HIIT, or a combination of these decreased risk factors in overweight/obese children [8,9,10]. However, it is known that nowadays children and adolescents spend a great part of their time in front of the screens [11], making their daily lives closer and closer to the virtual environment [12]. There are virtual reality alternatives to the screens that require the execution of body movements to advance in the game, such as exergaming (EXG) [13], which is becoming a novel therapy alternative to increase physical activity levels in children with overweight and obesity [14] and is an effective treatment to improve the health status of children and adolescents with overweight/obesity [15].

In a quasi-experimental study conducted by Marsigliante et al. [16] in children, a 6-month EXG intervention was reported as an effective strategy to address childhood obesity, improve physical skills, and increase students’ enjoyment, encouraging long-term physical activity adherence. In terms of morphological variables in a meta-analysis conducted in normal-weight children, EXG reported significant decreases in BMI (*p* = 0.000), with no significant decrease in body fat percentage (*p* = 0.059) in favor of EXG compared to inactive CG in 18-week interventions [17]. The meta-analysis in children with overweight/obesity reported that EXG interventions did not show significant decreases in BMI (*p* > 0.05) compared to inactive CG [18]. Some systematic reviews regarding cardiovascular and biochemical parameters show unclear results in children and adolescents with EXG interventions. A systematic review by Pereira et al. [19] in adolescents with obesity and autism showed no changes in SBP and diastolic blood pressure (DBP) in favor of EXG interventions compared to CG. However, a Chen et al. [20] meta-analysis in adults undergoing cardiac rehabilitation showed significant decreases in LDL-cholesterol (*p* = 0.01) in favor of EXG interventions compared to inactive CG.

Considering that the technology is widely used in both children and adolescents, it can be an effective tool for health status markers in this age group [13]. This is why non-immersive virtual reality applied to body movement such as EXG has shown some positive effects on morphology, biochemical markers, and blood pressure, but the evidence remains inconsistent, especially with regard to cardiovascular outcomes in children and adolescents with obesity [17,18,19]. Thus, this systematic review and meta-analysis seeks to clarify the effects of EXG compared to CG on morphological variables, biochemical parameters, and blood pressure in children and adolescents with overweight/obesity.

## 2. Methods

### 2.1. Protocol and Registration

This systematic review followed the PRISMA principles [21]. The protocol is registered with PROSPERO (the International Prospective Register of Systematic Reviews; ID code: CRD42024626992).

### 2.2. Eligibility Criteria

The original, peer-reviewed papers published up until September 2024 that were not limited by language or publication date satisfied the inclusion requirements for this systematic review with meta-analysis. Conference abstracts, books and book chapters, editorials, letters to the editor, protocol records, reviews, case studies, and trials were among the resources that were not included. Furthermore, the PICOS (population, intervention, comparator, outcome, and study design) framework was used to do a systematic review (see Table 1).

**Table 1:** Selection criteria used in the systematic review.

Category	Inclusion Criteria	Exclusion Criteria
Population	Children or adolescents are considered to be participants under 18 years of age, according to UNICEF [22], and without distinction of sex, who are overweight/obese with a body mass index equal to or greater than 25.0 kg/m^2^ according to the World Health Organization [23].	People over 18 have a body mass index below 25.0 kg/m^2^ [23].
Intervention	Interventions use EXG or active non-immersive video games (i.e., Wii Sports, balance, and Fit, Switch Sports, Kinect Sports, Adventure and Your Shape, Sports Champions Move) for 4 weeks or more.	Interventions that do not use EXG or semi-immersive or immersive EXG as an intervention. There are no details of the intervention procedure.
Comparator	Interventions with active or inactive control groups.	Studies with no control groups or with inactive control groups.
Outcome	At least one assessment of morphological variables (i.e., body mass index, waist circumference, body composition), biochemical parameters (i.e., total cholesterol, HD-cholesterol, LDL-cholesterol, triglycerides, glucose), and blood pressure (systolic and diastolic blood pressure) before and after.	Lack of baseline data and/or follow-ups.
Study design	Randomized controlled trials, with pre- and post-assessment.	Non-randomized controlled trials, cross-sectional, retrospective, and prospective studies.

EXG: exergaming. UNICEF: United Nations Children’s Fund.

### 2.3. Information Search Process and Databases

Using five generic databases—PubMed, Medline, CINAHL Complete, Scopus, and Web of Science (core collection)—the search was carried out from February to September of 2024. Free language phrases pertaining to EXG, morphological characteristics, biochemical parameters, and blood pressure in children and adolescents who are overweight or obese were used by the US National Library of Medicine Medical Subject Headings (MeSH). The search string used was as follows: (“exergames” OR “exergaming” OR “active video games” OR “virtual reality” OR “Nintendo Wii” OR “Kinect” OR “PlayStation” OR “Nintendo Switch”) AND (“body composition” OR “body fat” OR “fat-free mass” OR “fat mass” OR “muscle mass” OR “body mass index” OR “nutritional status” OR “anthropometry”) AND (“biomarkers” OR “inflammation” OR “lipid metabolism” OR “lipid metabolism disorders” OR “diabetes mellitus” OR “glycemic control” OR “C-reactive protein” OR “kidney function tests” OR “liver function tests” OR “electrolytes” OR “pancreatic function tests”) AND (“children” OR “child” OR “school children” OR “schoolchildren” OR “childhood” OR “young” OR “youth” OR “adolescents” OR “teen” OR “high school students”) AND (“obesity” OR “pediatric obesity” OR “obesity morbid” OR “overweight”). Two separate experts were consulted over the included articles and the inclusion and exclusion criteria in order to help find more pertinent studies. The experts had to meet two criteria: (i) possess a doctorate in sports science, and (ii) have peer-reviewed papers on physical performance in different population groups and/or physical performance published in journals using Journal Citation Reports^®^’s impact factor. To prevent bias in their searches, we kept our search approach a secret from experts. Following these procedures, on 30 September 2024, we looked through a database for pertinent retractions or errata pertaining to the works on the list.

### 2.4. Studies Selection and Data Collection Process

The studies were exported using the EndNote reference manager (version X9, Clarivate Analytics, Philadelphia, PA, USA). Separate searches were carried out by JPC and JHM, who also looked at titles and abstracts, removed duplicates, and read the entire texts. No discrepancies have been found as of yet. The process was repeated for searches inside reference lists and referrals from outside experts. After reviewing the texts of potentially appropriate papers, the justification for excluding those that did not meet the selection criteria was revealed.

### 2.5. Methodological Quality Assessment

The methodological quality of the selected studies was evaluated using TESTEX, a tool for exercise-based intervention studies [24]. TESTEX findings were one possible exclusion criterion assessed [24]. According to Smart, Waldron, Ismail, Giallauria, Vigorito, Cornelissen, and Dieberg [24], there is a 15-point rating system (5 points for study quality and 10 points for reporting). A third author (ICC) acted as a referee for cases that were on the borderline and required additional validation from another author (PVB), while two authors (JPC, JHM) completed this process independently.

### 2.6. Data Synthesis

From the chosen studies, the following information was gathered and examined: (i) author and publication year; (ii) nation of origin; (iii) research methodology; (iv) the sample’s starting health; (v) the number of participants in the intervention and control groups; (vi) the sample’s average age; (vii) the activities engaged in during the EXG and regular physical activity; (viii) training volume (total duration, weekly frequency, and time per session); (ix) training intensity; (x) biochemical parameters (total cholesterol, HD-cholesterol, LDL-cholesterol, triglycerides, glucose), blood pressure (SBP and DBP), morphological variables (BMI, waist circumference, body fat percentage, and fat-free mass), and (xi) the primary findings of the studies.

### 2.7. Risk of Bias in Individual Studies

A third researcher (EVC) examined the findings after two independent researchers (JPC and JHM) assessed the included studies’ risk of bias version 2 (RoB 2). This review was based on the guidelines for RCTs in the Cochrane Handbook for Systematic Reviews of Interventions [25]. The risk of bias was classified as “high,” “low,” or “some concerns” depending on the randomization process, deviations from the planned interventions, missing outcome data, outcome evaluation, and the choice of the reported result.

### 2.8. Summary Measures for Meta-Analysis

The study methodology includes meta-analysis; complete information is accessible at PROSPERO (registration code: CRD42024626992). Meta-analyses were only performed in the present case when ≥3 studies were available [26]. Effect sizes (ES; Hedge’s g) for each attribute of morphological variables, biochemical parameters, and blood pressure in the EXG and control groups were calculated using the pretraining and post-training mean and SD (standard deviation) for each dependent variable. Data were standardized using the change score SD. The ES values are presented with 95% confidence intervals (95%CIs). Calculated ES were interpreted using the following scale: trivial: <0.2; small: 0.2–0.6; moderate: >0.6–1.2; large: >1.2–2.0; very large: >2.0–4.0; and extremely large: >4.0 [27]. The random effects model was used to account for differences between studies that might affect the effect of EXG. Comprehensive Meta-analysis software (Version 2.0; Biostat, Englewood, NJ, USA). Statistical significance was set at *p* ≤ 0.05 and used to perform these calculations [28]. In each trial, the random effects model (Der Simonian-Laird approach) was used to calculate and pool the SMD and MD of BMI, waist circumference, percent body fat, fat-free mass, total cholesterol, HD cholesterol, LDL cholesterol, triglycerides, glucose, SBP, and DBP (EXG vs. CG). The fundamental premise of the random-effects model is that genuine effects (interventions, duration, among others) vary throughout studies and that samples are selected from populations with varying effect sizes. The data pooled if at least three studies showed the same results [26].

Heterogeneity between trial results was tested with a Cochran’s Q test (Morris, 2008) and I^2^ statistic. I^2^ values of <25%, 25–50%, and >50% represent small, medium, and large amounts of inconsistency [29]. Egger regression tests were performed to detect small study effects and possible publication bias [30].

### 2.9. Moderator Analyses

A random-effects model and independent computed single-component analysis were used to select potential sources of heterogeneity that could impact the training effects a priori.

### 2.10. Factor Analysis of Single Training

Single training factor analyses were calculated for age, program duration (number of weeks), and training frequency (number of sessions per week) based on the reported influence of these variables on the adaptations generated to training in children and adolescents with overweight/obesity [31].

When appropriate, subgroup analyses and single training factor analyses were divided using the median split technique [15]. The median was calculated if at least 2 studies provided data for a given moderator [32]. Of note, when 2 experimental groups with the same information for a given moderator were included in a study, only one of the groups was considered in order to avoid an undue influence on the median calculation. In addition, to minimize heterogeneity, instead of using a global median value for a given moderator (e.g., median training frequency derived from all included studies), median values were calculated using only those studies that provided data for the outcome being analyzed.

### 2.11. Certainty of Evidence

Based on their evaluation of the GRADE scale, studies were classified as having high, moderate, low, or very low confidence [33]. All analyses started with a high degree of certainty because they contained papers with randomized controlled trial designs, and they were degraded if there were issues with the risk of publication bias, consistency, accuracy, precision, directness of results, or risk of bias [33]. The studies were examined independently by two authors (JPC and JHM), and any discrepancies were resolved by consensus with a third author (EVC).

## 3. Results

### 3.1. Study Selection

**Figure 1:**
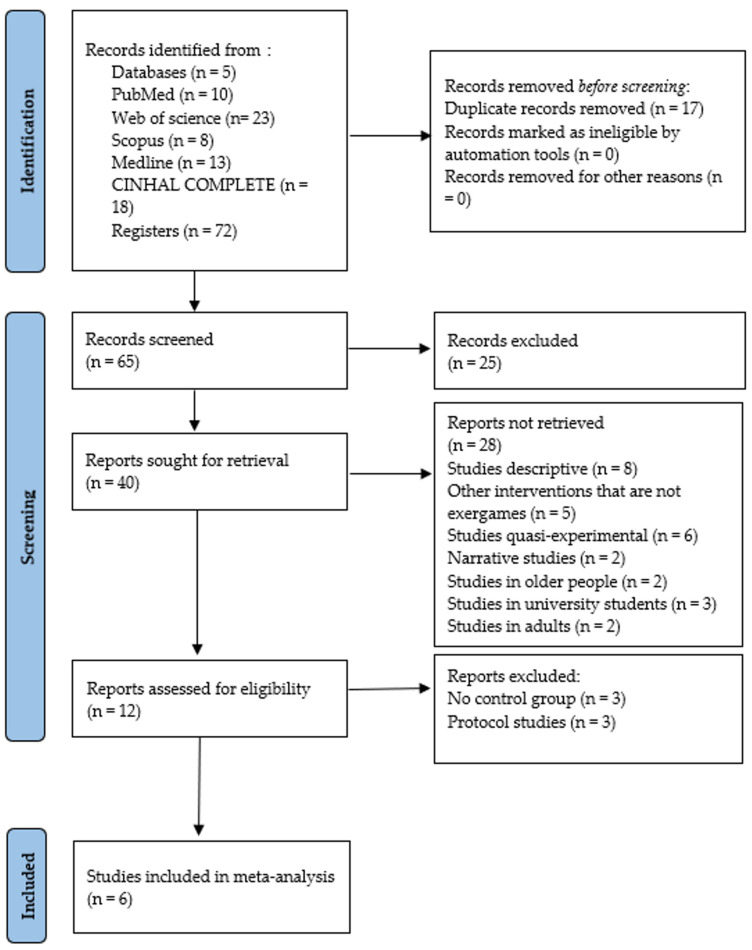
Flowchart of the review process.

Figure 1 details the search process for the studies. A total of 72 records were found. Subsequently, duplicates were eliminated, and the studies were filtered by selecting the title, abstract, and keywords, resulting in 65 references. In the subsequent analysis phase, 25 articles were excluded because the texts did not meet the search criteria, leaving 40. Subsequently, 28 were excluded: 8 descriptive studies, 5 other types of interventions other than EXG, 6 quasi-experimental studies, 2 narrative studies, 2 studies in older people, 3 studies in university students, and 2 studies in adults. After this process, 12 potential studies remained, of which 3 were excluded because they did not have a CG and 3 protocol studies; 6 studies met all the selection criteria [14,34,35,36,37,38].

### 3.2. Methodological Quality

The 6 selected studies were analyzed using the TESTEX scale (Table 2). All studies achieved a score equal to or above 60% on the scale, namely 8/15 [38], 9/15 [35], 10/15 [37], 11/15 [36], and 13/15 [14,34], indicating moderate to high methodological quality, so no study was excluded from the systematic review.

**Table 2:** Study quality assessment according to the TESTEX scale.

Study	Eligibility Criteria Specified	Randomly Allocated Participants	Allocation Concealed	Groups Similar at Baseline	Assessors Blinded	Outcome Measures Assessed >85% of Participants *	Intention to Treat Analysis	Reporting of Group Statistical Comparisons	Point Measures and Measures of Variability Reported **	Activity Monitoring in Control Group	Relative Exercise Intensity Reviewed	Exercise Volume and Energy Expended	Overall TESTEX#
Abedelmalek, Aloui, Denguezli Bouzgarou, Adam, Souissi and Chtourou [36]	Yes	Yes	No	Yes	No	Yes (2)	No	Yes	Yes (2)	Yes	Yes	Yes	11/15
Staiano, Beyl, Guan, Hendrick, Hsia, and Newton [14]	Yes	Yes	Yes	Yes	Yes	Yes (2)	Yes	Yes	Yes (2)	Yes	No	Yes	13/15
Staiano, Marker, Beyl, Hsia, Katzmarzyk and Newton [34]	Yes	Yes	Yes	Yes	Yes	Yes (2)	Yes	Yes	Yes (2)	Yes	No	Yes	13/15
van Biljon, Longhurst, Shaw and Shaw [35]	Yes	Yes	No	Yes	No	Yes (1)	No	Yes	Yes (2)	Yes	No	Yes	9/15
Adamo, Rutherford and Goldfield [37]	Yes	Yes	No	Yes	No	Yes (2)	No	Yes	Yes (1)	Yes	Yes	Yes	10/15
Murphy, Carson, Neal, Baylis, Donley, and Yeater [38]	Yes	Yes	No	Yes	No	Yes (1)	No	Yes	Yes (1)	Yes	No	Yes	8/15

* Three points are possible: one point if adherence >85%, one point if adverse events were reported, and one point if exercise attendance was reported. ** Two points possible: one point if the primary outcome is reported, one point if all other outcomes were reported. # total out of 15 points. TESTEX: Tool for assessing study quality and reporting in exercise.

### 3.3. Risk of Bias

**Figure 2:**
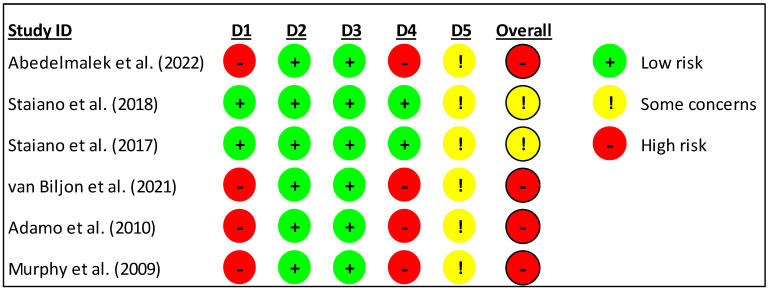
Risk of bias within studies. Legends: D1: randomization process; D2: deviations from the intended interventions; D3: missing outcome data; D4: measurement of the outcome; D5: selection of the reported result.

**Figure 3:**
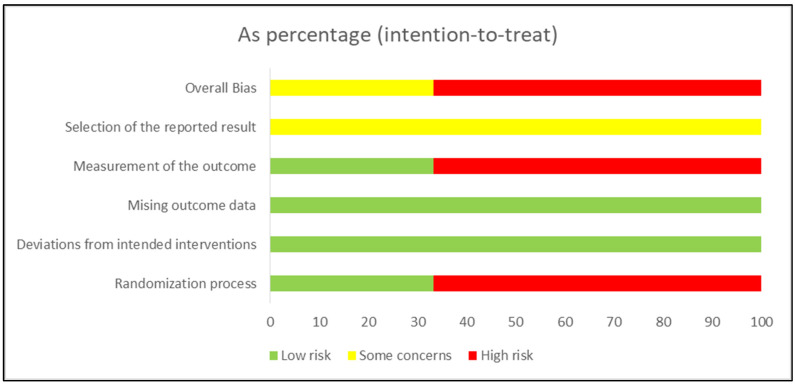
Risk of bias summary: Review the authors; judgments about each risk of bias item in each included study.

The risk of bias was high for 4 studies [35,36,37,38] and 2 studies with some concerns [14,34]. In the randomization process, 2 studies were low risk [14,34], and 4 studies were high risk [35,36,37,38]. While in deviations from the intended interventions, all studies show a low risk [14,34,35,36,37,38]. All studies showed low risk in the missing outcome data [14,34,35,36,37,38]. In measuring the outcome, 2 studies were low risk [14,34], and 4 studies were high risk [35,36,37,38]. While selecting the reported results, all studies showed some concerns [14,34,35,36,37,38]. The risk of bias summary is presented in Figure 2, and the risk of bias graph is presented in Figure 3.

### 3.4. Studies Characteristics

The variables analyzed in the 6 selected studies are listed in Table S1. Three studies were in the United States of America [14,34,38], 1 in Tunisia [36], 1 in Canada [37], and 1 in South Africa [35]. The 6 studies selected were randomized controlled trials [14,34,35,36,37,38].

### 3.5. Sample Characteristics

Six studies reported participant numbers ranging from 21 to 46 [14,34,35,36,37,38]. As a result, the combined sample size across all these studies consisted of 167 children and 26 adolescents with overweight/obesity, with a mean age of 13.91 years [14,34,35,36,37,38].

### 3.6. Dosing and Conducted Interventions

The interventions lasted between 4 and 24 weeks, with a training frequency of 2 to 5 days per week and session durations ranging from 30 to 60 min each [14,34,35,36,37,38]. Only 1 study did not report the training intensity [35]. Additionally, just 1 study measured intensity using the CERT (child effort rating table) every 2 min, with 1 representing very low intensity and 10 indicating high intensity [38]. The use of video game consoles varied across studies. The Xbox Kinect was used in 2 studies [14,34], and PlayStation 2 [37,38]. The Nintendo Wii was used in 1 study [35], while 1 study did not specify the type of console used [36]. Four categories of EXG interventions were used: (i) dance sessions, (ii) cooperative EXG sessions, (iii) boxing and hula hoop sessions, and (iv) cycling static sessions. Dance sessions were used in 3 studies [14,34,38]. Cooperative EXG sessions were employed in 1 study [36], while boxing and hula hoop sessions were featured in another [35]. Static cycling sessions were used in 1 study [37].

### 3.7. Meta-Analysis Results

The overall effects of EXG on morphological variables, biochemical parameters, and blood pressure are shown in Table 3. Forest plots are shown in Figures S1–S10. There were moderate to large significant effects (*p* < 0.05) in favor of EXG in BMI, waist circumference, and SBP (ES = 0.45 to 0.64). While in body fat percentage and DBP, no significant differences were reported (*p* > 0.05) with small effect sizes (ES = 0.003 to 0.08). Similarly, in the biochemical parameters total cholesterol, LDL-cholesterol, HDL-cholesterol, triglycerides, and glucose, no significant differences were reported (*p* > 0.05) with small to moderate effect sizes (ES = −0.20 to 0.42).

### 3.8. Moderator Analyses

Moderator analyses were considered, given that ≥2 studies per moderator were available. In total, 16 subgroup analyses were performed for training factors: total cholesterol, LDL-cholesterol, HDL-cholesterol, and triglycerides (age, duration of intervention, and frequency of training). The analyses are summarized below, with full descriptions presented in Figures S1–S10.

### 3.9. Certainty of Evidence

The results of the certainty of evidence range from down to moderate and do not allow definitive recommendations on the use of EXG interventions on morphological variables, biochemical parameters, and blood pressure compared to CG in children and adolescents with overweight/obesity (Table 4).

### 3.10. Adverse Events and Adherence

Only one study reported adverse events when performing an EXG intervention, which was mild hematomas (hand lacerations and back pain) [34]. Regarding adherence, three studies showed 100% adherence [35,36,38]. Other studies showed adherence of 94% [34], 88% [37], and 62% [14], respectively. In all three studies, no injuries were reported during the interventions, and no health problems were reported, the main reason being lack of time because not all sessions were completed.

## 4. Discussion

### 4.1. Body Mass Index (BMI)

In the present meta-analysis, significant decreases in BMI were found in favor of EXG concerning CG. Similar to that reported in a meta-analysis by Comeras-Chueca, Marin-Puyalto, Matute-Llorente, Vicente-Rodriguez, Casajus, and Gonzalez-Aguero [17] of randomized and non-randomized controlled trial studies in normal-weight children, reporting significant decreases in BMI (*p* = 0.000) in favor of EXG interventions lasting more than 18 weeks vs. inactive CG. Different from that reported by Erçelik and Çağlar [18] in a meta-analysis of randomized controlled trial studies conducted in children and adolescents with overweight/obesity, where there were no significant differences (*p* > 0.05) in BMI between EXG vs. active/inactive CG. The differences in the findings of the mentioned studies can be attributed to the dietary habits of the participants since not all the studies controlled the diet of the participants during the interventions; this is relevant since it has been reported that physical activity together with a balanced intake of macronutrients helps to reduce the BMI [39]. In this sense, it has been reported that the reduction of the consumption of foods high in saturated fats, sodium, and sugars when combined with physical activity can positively change the BMI of children and adolescents overweight [40]. On the other hand, it is essential to consider that the energy metabolism of skeletal muscle may differ depending on the console and EXG used during the interventions [41]. In particular, EXGs that involve lower limb muscle groups are more demanding since they involve greater muscle mass, leading to greater energy expenditure than those that involve only the upper limbs [42]. For example, Calcaterra et al. [43] have reported that the most actively exercising games are dance simulation products (e.g., Dance Dance Revolution^®^, DDR) and Wii boxing^®^, which should be encouraged to combat or prevent pediatric obesity. Only one study in the overall meta-analysis reported no significant decreases in BMI [35]. This could be attributed to the duration of the intervention since it was the shortest (6 weeks) compared to the rest of the meta-analyzed studies, which ranged from 10 to 24 weeks. Therefore, based on our meta-analysis, at least 10 weeks of EXG training might be necessary to induce significant improvements in BMI in children and adolescents with overweight/obesity. On the other hand, the study by van Biljon, Longhurst, Shaw, and Shaw [35] did not report the intensity of exercise during EXG. Relevant aspect, given that intensity is key to generating physical and physiological adaptations in people; not knowing the intensity at which the intervention was carried out limits the possibilities of a greater comparison with the rest of the studies.

### 4.2. Waist Circumference

In the present meta-analysis, significant decreases in waist circumference were reported in favor of EXG over CG. Similar to that reported in a systematic review by Lamboglia et al. [44] of randomized and non-randomized controlled trial studies in children with obesity, mentioning that EXG interventions significantly reduced waist circumference compared to inactive CG. This is different from that reported by Oliveira et al. [45] in a meta-analysis of randomized controlled trial studies in children and adolescents with obesity, where they found no significant effect (*p* > 0.05) in favor of EXG on waist circumference compared to active/inactive CG. However, Oliveira, Pinto, Saraiva, Tebar, Delfino, Franco, Silva, and Christofaro [45] mentioned that pooled estimates for waist circumference showed very low quality of evidence, indicating high uncertainty in these estimates and requiring further studies to reach a definitive conclusion. In our overall meta-analysis, only the study by Abedelmalek, Aloui, Denguezli Bouzgarou, Adam, Souissi, and Chtourou [36] reported no significant improvements in waist circumference in favor of EXG. In this regard, it is important to mention that the intensity of EXG was not controlled in this study and that it was the intervention with the shortest duration (4 weeks) compared to the study by Adamo, Rutherford, and Goldfield [37] and Staiano, Beyl, Guan, Hendrick, Hsia, and Newton [14], which lasted 10 and 12 weeks, respectively. In this sense, EXG interventions, similarly to conventional physical activity, may require a considerably longer time to positively modify morphological variables [15]. Furthermore, it has been suggested that both the duration and intensity of training are vital variables for the efficacy of EXG programs.

### 4.3. Body Fat Percentage

Concerning body fat percentage, no significant decreases were reported in favor of EXG regarding CG. Results similar to those presented by Comeras-Chueca, Marin-Puyalto, Matute-Llorente, Vicente-Rodriguez, Casajus, and Gonzalez-Aguero [17] in children with normal weight did not find significant decreases (*p* = 0.059) in favor of EXG in body fat percentage compared to CG. Similar to the BMI and waist circumference variables, certain limitations in studies that have assessed the effects of EXG on body composition in obese children and adolescents should be considered [17]. For example, the inclusion of uncontrolled trials, the inclusion of children and adolescents with medical conditions, and the different consoles and video games used. Furthermore, as mentioned above, interventions with EXG require dietary control in participants to optimize their results and generate positive changes in the body composition of overweight children and adolescents [15]. Developing healthy eating habits early is essential since childhood obesity is associated with negative differences between energy consumption and expenditure, resulting in a positive energy balance and, consequently, an increase in body fat percentage [44]. In this sense, adopting a healthy lifestyle through balanced nutrition and physical activity can considerably improve the body composition of overweight children and adolescents.

### 4.4. Biochemical Parameters (HDL-Cholesterol, LDL-Cholesterol, Total Cholesterol, and Triglycerides)

The present meta-analysis showed no significant reduction in favor of EXG in HDL cholesterol compared with active/inactive CG. Similar to that presented, in the meta-analysis of randomized controlled trial studies conducted by Chen, Cao, Xu, Zhu, Guan, and Ming [20] in adults undergoing cardiac rehabilitation, there were no significant changes (*p* = 0.62) in HDL-cholesterol in favor of EXG compared to inactive CG. Similarly, in a meta-analysis conducted by Yao et al. [46] in adults with diabetes, there was no significant change (*p* = 0.61) in HDL-cholesterol in favor of interventions with EXG compared to CG. Secondly, there were no significant decreases in favor of EXG in LDL-cholesterol compared to CG. Similarly, in his meta-analysis carried out in adults with diabetes mellitus with overweight. Lim et al. [47] showed no significant changes (*p* = 0.96) in LDL-cholesterol in favor of EXG for CG. On the contrary, a meta-analysis conducted by Chen, Cao, Xu, Zhu, Guan, and Ming [20] showed significant decreases in LDL-cholesterol (*p* = 0.01) in favor of EXG interventions compared to inactive CG. Total cholesterol showed no significant decreases in favor of EXG about CG, contrary to that reported in a meta-analysis by Erçelik and Çağlar [18], showing significant decreases (*p* < 0.0001) in total cholesterol in favor of EXG compared to CG. Similarly, a meta-analysis conducted by Chen, Cao, Xu, Zhu, Guan, and Ming [20] showed significant decreases in total cholesterol (*p* = 0.004) in favor of EXG interventions compared to inactive CG. Finally, triglycerides show no significant decreases in favor of EXG compared to CG in triglycerides. Similarly, Chen, Cao, Xu, Zhu, Guan, and Ming [20] found no significant changes (*p* = 0.74) in triglycerides in favor of EXG compared to inactive CG in adults undergoing cardiac rehabilitation. Similarly, in a meta-analysis conducted by Yao, Han, Yang, Chen, Yan, and Cheng [46], there was no significant change (*p* = 0.89) in triglycerides in favor of interventions with EXG compared to CG.

Physical activity and a healthy diet are crucial for maintaining a normal lipid profile and reducing the cardiovascular risk generated by overweight [48]. Low HDL levels are often accompanied by high triglyceride levels, insulin resistance, and abdominal obesity [49]. Importantly, the impact of EXG on the lipid profile depends on multiple factors, including the type of EXG, the console used, the muscle groups involved, duration, and intensity, which should be accompanied by a healthy diet, although the exact mechanisms of the beneficial impact of EXG on the lipid profile remain unclear.

### 4.5. Blood Pressure

Our meta-analysis reported significant improvements for SBP with EXG. Unlike that mentioned by Lim, Ho, and Goh [47] in a meta-analysis of randomized controlled trial studies in adults with diabetes mellitus, where no significant changes were found in SBP (*p* = 0.12) and DBP (*p* = 0.28) in favor of EXG compared to CG. Similarly, Pereira, Morais, Gabriel, Claumann, Helal, Roever, and Farias [19], a systematic review of randomized controlled trial studies conducted in adolescents with obesity and autism, showed no changes in SBP and DBP in favor of interventions with EXG compared to CG. In this regard, the literature has suggested that intensity is a key factor in generating improvements in SBP and DBP [50]. Studies that did not report improvements in blood pressure may require higher intensity (>70% of maximal heart rate) EXG to induce favorable adaptations in SBP and DBP [50]. Specifically, higher intensity may generate a higher oxygen demand in the involved muscles during exercise, leading to increased blood flow through the vessel and promoting increased nitric oxide release [50]. Furthermore, greater involvement of muscle groups during exercise may physiologically generate a greater release of vasoactive substances (nitric oxide, prostaglandins) caused by a greater increase in blood flow, which may be beneficial in the long term in children and adolescents with overweight/obesity [51]. Based on our meta-analysis, at least 24 weeks with a frequency of 3 times per week and 60-min sessions using EXG at moderate and vigorous intensity may be necessary to induce improvements in SBP and DBP [52,53]. In this context, it is imperative to motivate children and adolescents to participate in EXG programs, since it seems to be more important to increase their physical activity levels rather than reduce their overall sedentary time to improve cardio-metabolic health [52].

### 4.6. Glucose

In the present meta-analysis, there were no significant decreases in glucose in favor of EXG regarding CG. Similarly, Pereira, Morais, Gabriel, Claumann, Helal, Roever, and Farias [19] in a systematic review showed no decreases (*p* > 0.05) in glucose in favor of interventions with EXG compared to CG. Similarly, in a meta-analysis conducted by Yao, Han, Yang, Chen, Yan, and Cheng [46], glucose had no significant decreases (*p* = 0.83) in favor of EXG interventions compared to CG. The literature has reported that physical activity is a good alternative for controlling blood glucose levels in overweight patients [54]. Furthermore, improvements have been reported through aerobic exercise in glycemic control and cardiovascular risk factors, through an increase in skeletal muscle capitalization and blood flow, muscle levels of glucose transporter type 4, hexokinases, and glycogen synthase activities [55]. Likewise, strength training has also reported positive effects, including an increase in the number of glucose transporter proteins, increased total muscle mass, and an increase in the number of insulin receptors in muscle cells [54]. Therefore, based on the findings of our meta-analysis, further research is needed to elucidate the possible mechanisms that influence minor improvements in glucose levels through EXG since factors such as duration, intensity, and type of EXG could be relevant in these adaptations [19].

### 4.7. Subgroup Analysis by Age

In <15 years of age, significant differences (*p* = 0.006) were found in favor of EXG in total cholesterol (3 experimental groups; ES = 0.56; 95% CI = 0.16 to 0.96; within-group I^2^ = 0.00%), LDL-cholesterol (*p* = 0.01) (3 experimental groups; ES = 0.63; 95% CI = 0.22 to 1.05; within-group I^2^ = 4.33%), HDL-cholesterol (*p* = 0.01) (3 experimental groups; ES = 0.51; 95% CI = −0.41 to 0.11; within-group I^2^ = 0.00%), triglycerides (*p* = 0.000) (3 experimental groups; ES = 0.82; 95% CI = 0.41 to 1.23 within-group I^2^ = 0.00%). This finding is relevant, given that our meta-analysis indicates that children under 15 years of age can improve all biochemical parameters related to the lipid profile through EXG interventions. In this context, the improvements observed in our meta-analysis could be attributed to a decrease in the concentrations of CRP, TNF-α, and IL-1β [49]. CRP is an acute-phase protein produced by the liver in response to injury and/or infection. Increased levels have been associated with the development of atherosclerosis, ischemic attacks, and hemorrhagic strokes [49]. Physiologically, CRP binds to apolipoprotein B (apoB) containing LDL and VLDL, modifying lipid metabolism. In vitro, CRP has been reported to bind to phospholipids in liposomes and cell membranes, altering triacylglycerol, cholesterol, LDL, and fatty acid levels [39]. On the other hand, it has been reported that EXG can decrease serum levels of TNF-α, which are significantly high in the adipose tissue of obese individuals [15,17,18]. Elevated levels of TNF-α induce insulin resistance and decrease the expression of glucose transporters GLUT-4 [49]. Considering the low levels of physical activity performed by children and adolescents with overweight/obesity, promoting physical activity through EXG is a valuable tool for this population. Since, as mentioned in previous sections, EXG can increase enjoyment, which has been identified as a key factor in generating greater long-term adherence compared to conventional physical activity [16].

In ≥15 years of age there were no significant differences in total cholesterol (3 experimental groups; ES = 0.19; 95% CI = −1.10 to 1.50; within-group I^2^ = 84.8%), LDL-cholesterol (*p* = 0.65) (3 experimental groups; ES = −0.17; 95% CI = −0.96 to 0.60; within-group I^2^ = 60.4%), HDL-cholesterol (*p* = 0.55) (3 experimental groups; ES = −0.31; 95% CI = −0.71 to 1.33; within-group I^2^ = 76.5%), triglycerides (*p* = 0.74) (3 experimental groups; ES = −0.15; 95% CI = −1.07 to 0.77 within-group I^2^ = 71.3%). Although we did not find improvements with EXG for this subgroup, there was also no worsening of the lipid profile in this population, so the implementation of training programs with EXG is at least not harmful for this population. It is important to mention that studies with adolescents with an average age of 15 to 17 years are limited. Our meta-analysis only incorporated two studies with EXG in this age range. The studies had a duration of 4 and 12 weeks prescribing an intensity of 50 to 70% of the maximum heart rate, which is not different from the studies meta-analyzed for the age group <15 years. In this sense, further research is needed to identify the optimal intensity and volume in adolescents ≥15 years to induce improvements in their lipid profile.

### 4.8. Subgroup Analysis by Training Duration

In <12 weeks we found significant differences in favor of EXG in total cholesterol (*p* = 0.02) (3 experimental groups; ES = 0.69; 95% CI = 0.09 to 0.91; within-group I^2^ = 0.00%), without presenting significant differences in LDL-cholesterol (*p* = 0.09) (3 experimental groups; ES = 0.53; 95% CI = −0.09 to 1.15; within-group I^2^ = 8.53%), HDL-cholesterol (*p* = 0.11) (3 experimental groups; ES = −0.52; 95% CI = −1.16 to 0.12; within-group I^2^ = 13.9%), triglycerides (*p* = 0.13) (3 experimental groups; ES = 0.45; 95% CI = −0.13 to 1.04 within-group I^2^ = 0.00%). On the other hand, in ≥12 weeks we did not find significant differences in favor of EXG in total cholesterol (*p* = 0.56) (3 experimental groups; ES = 0.22; 95% CI = −0.54 to 0.98; within-group I^2^ = 77.4%), LDL-cholesterol (*p* = 0.67) (3 experimental groups; ES = 0.17; 95% CI = −0.63 to 0.98; within-group I^2^ = 80%), HDL-cholesterol (*p* = 0.96) (3 experimental groups; ES = −0.01; 95% CI = −0.83 to 0.79; within-group I^2^ = 80%), triglycerides (*p* = 0.43) (3 experimental groups; ES = 0.40; 95% CI = −0.59 to 1.40 within-group I^2^ = 86.2%). In this regard, it is well documented that physical activity is important for regulating cholesterol metabolism in overweight and obese individuals [16]. However, improvements in total cholesterol may be subject to acute interfering factors such as diet [48,56]. In this regard, the differences found in total cholesterol between studies < 12 weeks and ≥12 weeks may be attributed to the fact that not all studies controlled or prescribed a diet to participants during the interventions. Dietary habits may play a key role in inducing improvements in the lipid profile when performed in conjunction with physical activity interventions [39]. For example, reducing the consumption of processed foods high in fat, sodium, and sugars in combination with physical activity may induce improvements in the lipid profile of children and adolescents with overweight/obesity [39]. Therefore, it is imperative that future studies control the diet of participants and include dietary advice in their interventions.

### 4.9. Subgroup Analysis by Training Frequency

In <3 sessions per week we found no significant differences in favor of EXG in total cholesterol (*p* = 0.51) (3 experimental groups; ES = 0.27; 95% CI = −0.56 to 1.12; within-group I^2^ = 77.9%), LDL-cholesterol (*p* = 0.45) (3 experimental groups; ES = 0.37; 95% CI = −0.61 to 1.35; within-group I^2^ = 83.2%), HDL-cholesterol (*p* = 0.81) (3 experimental groups; ES = −0.11; 95% CI = −1.07 to 0.84; within-group I^2^ = 82.6%), triglycerides (*p* = 0.60) (3 experimental groups; ES = 0.24; 95% CI = −0.68 to 1.18 within-group I^2^ = 81.8%). On the other hand, in ≥3 sessions per week we only identified significant differences in favor of EXG in triglycerides (*p* = 0.03) (3 experimental groups; ES = 0.70; 95% CI = −0.04 to 1.36; within-group I^2^ = 35%), without presenting significant differences in total cholesterol (*p* = 0.08) (3 experimental groups; ES = 0.53; 95% CI = −0.08 to 1.15; within-group I^2^ = 27%), LDL-cholesterol (*p* = 0.35) (3 experimental groups; ES = 0.24; 95% CI = −0.26 to 0.75; within-group I^2^ = 0.00%), HDL-cholesterol (*p* = 0.13) (3 experimental groups; ES = −0.39; 95% CI = −0.91 to 0.12 within-group I^2^ = 0.00%). To our knowledge, this is the first systematic review that includes a subgroup meta-analysis for the variables of age, duration, and frequency of training. Our findings for training frequency suggest that ≥3 sessions per week may be necessary to induce improvements in the lipid profile, specifically in triglyceride values, in children and adolescents with overweight/obesity. Lower training frequencies (<3 sessions per week) may not be a sufficient stimulus to generate improvements in these variables, although they depend on key variables such as the duration and intensity of the interventions. In this context, it has been suggested that EXG interventions may require a higher frequency to positively affect lipid profile variables in children and adolescents with overweight/obesity [15]. However, it is important to consider that higher training frequencies may compromise the feasibility and adherence of the interventions in this population. Therefore, based on our findings and current evidence, a frequency of three times per week may be an optimal starting point to induce health improvements in children and adolescents with overweight/obesity, while increasing motivation and enjoyment of physical activity through EXG.

### 4.10. Certainty of Evidence

The certainty of evidence for the studies analyzed varies from moderate to low for morphological variables, biochemical parameters, and blood pressure, which does not allow definitive recommendations on using EXG in children and adolescents with overweight/obesity. Similar to that reported by Liu et al. [57] in a systematic review of children analyzing the effects of EXG interventions on physical fitness and fundamental motor skills with a low certainty of evidence. Similarly, an overview by Hernandez et al. [58] in older people presents moderate to low certainty of evidence on physical performance variables for using EXG as an intervention in this age group.

### 4.11. Strengths and Limitations

Limitations include: (i) variability in the consoles used as well as in the games, which may result in different responses to EXG interventions [59]; (ii) not analyzing different types of virtual performance by EXG, which may differ in responses to interventions; (iii) the low number of RCTs may limit the robustness of the moderator outcomes (age, duration of intervention, and frequency of training) for subgroup analyses, as well as the low number of total samples included for meta-analysis. In the strengths, we found: (i) the methodological quality above 60% in the studies analyzed; (ii) the methodological processes that followed the PRISMA, PROSPERO, TESTEX, RoB 2, and GRADE scales; (iii) the use of 5 generic databases: PubMed, Medline, CINAHL Complete, Scopus, and Web of Science; (iv) performing meta-analysis of subgroups divided by age, duration of intervention, and frequency of training; (v) analyzing the response of the EXGs together on morphological variables, biochemical parameters, and blood pressure in children and adolescents with overweight/obesity. However, it is unclear whether these results are maintained in the long term. Therefore, we encourage investigators to use long-term follow-up evaluations to determine the sustainability of EXGs in treatments that aim to regulate alterations in cardiometabolic risk factors in children and adolescents with overweight/obesity.

### 4.12. Practical Applications

The study provides several practical applications, particularly in addressing childhood obesity through innovative interventions. Here are the key applications:

- EXG as a strategy for obesity management: the study highlights EXG as an effective strategy to combat childhood obesity. By integrating physical activity with gaming, EXG can enhance engagement and adherence among children and adolescents, making it a viable alternative to conventional physical activity programs.
- Improved biochemical parameters in children < 15 years of age: findings suggest that exercise with EXG can significantly improve lipid profiles, including reductions in total cholesterol, LDL-cholesterol, and triglycerides while increasing HDL cholesterol; this suggests that exercise with EXG can be incorporated into programs aimed at improving cardiovascular health in children and adolescents with overweight/obesity.
- Promotion of long-term physical activity: the study emphasizes the potential of EXG to increase enjoyment and motivation for physical activity, which is crucial for fostering long-term adherence to an active lifestyle; this can lead to sustained health benefits beyond the intervention period.
- Customizable intervention programs: given the variability in the types of games and consoles used, EXG interventions can be tailored to individual preferences and capabilities, enhancing their effectiveness and appeal to diverse populations.
- Integration with nutritional guidance: the study suggests that combining EXG with dietary control could optimize body composition and metabolic health outcomes. This integrated approach can be particularly beneficial in comprehensive weight management programs.
- Potential for school-based implementation: EXG can be easily implemented in school settings, providing a structured environment for regular physical activity; this can help reach a larger population of children and adolescents, contributing to public health efforts in reducing obesity rates.

## 5. Conclusions

EXG in general shows significant decreases in BMI, waist circumference, and SBP. While in specific outcomes in children and adolescents with overweight/obesity under 15 years of age, EXG significantly decreases total cholesterol, LDL-cholesterol, and triglycerides, with significant increases in HDL-cholesterol compared to active/inactive CG. However, the certainty of the evidence varies from moderate to low and, therefore, does not allow definitive recommendations on the use of EXG in children and adolescents with overweight/obesity.

## Figures and Tables

**Table 3:** Synthesis of the results of the included studies on the effects of exergaming on morphological variables, biochemical parameters, and blood pressure in children and adolescents with overweight/obesity.

Morphological Variables, Biochemical Parameters, and Blood Pressure	*n* ^a^	ES (95%CI)	*p*	*I*^2^ (%)	Egger’s Test (*p*)	RW (%)
Morphological variables						
BMI (kg m^−2^)	4, 4, 4, 155.	0.51 (0.01 to 1.01)	**0.04**	52.2	**0.09**	3.17 to 4.48
Waist circumference (cm)	3, 3, 3, 90.	0.64 (0.03 to 1.26)	**0.03**	52.8	**0.12**	3.12 to 3.85
Body fat percentage (%)	3, 3, 3, 90.	0.003 (−0.39 to 0.40)	0.99	0.00	**0.68**	6.38 to 10.5
Biochemical parameters						
Total cholesterol (mg/dl)	5, 5, 5, 173.	0.38 (−0.14 to 0.90)	0.15	63.7	0.02	2.30 to 3.15
LDL-cholesterol (mg/dl)	5, 5, 5, 173.	0.31 (−0.23 to 0.85)	0.26	66.6	0.01	2.07 to 2.88
HDL-cholesterol (mg/dl)	5, 5, 5, 173.	−0.20 (−0.77 to 0.36)	0.48	69.6	0.01	1.92 to 2.64
Triglycerides (mmol/L^−1^)	5, 5, 5, 173.	0.42 (−0.19 to 1.03)	0.17	72.9	0.005	1.77 to 2.27
Glucose (mg/dl)	3, 3, 3, 148.	0.40 (−0.13 to 0.94)	0.14	55,2	**0.10**	3.89 to 4.74
Blood pressure						
SBP (mmHg)	4, 4, 4, 143.	0.45 (0.12 to 0.78)	**0.007**	0.00	**0.44**	5.64 to 11.0
DBP (mmHg)	4, 4, 4, 143.	0.08 (−0.67 to 0.83)	0.83	79.9	0.02	1.53 to 1.76

Bolded *p*-values mean significant improvement (*p* < 0.05) in the experimental group after the exergaming intervention compared to the control group. ^a^ Data indicate the number of studies that provided data for analysis, the number of experimental groups, the number of control groups, and the total number of children and adolescents with overweight/obesity included in the analysis, respectively. Abbreviations: 95%CI = 95% confidence interval; ES = effect sizes (Hedge’s g); RW = relative weight of each study in the analysis.

**Table 4:** GRADE assessment for the certainty of evidence.

Assessment of Certainty							Number of Patients		Effect		Certainty	Importance
No. of Studies	Study Design	Risk of Bias	Inconsistency	Indirect Evidence	Vagueness	Other Considerations	Exergaming	Control Group	Relative (95% CI)	Absolute (95% CI)		
**Exergaming During Ramadan Intermittent Fasting Improve Body Composition as Well as Physiological and Psychological Responses to Physical Exercise in Adolescents with Obesity**												
1	Randomized trials	Very serious ^a^	It’s not serious	It’s not serious	It’s not serious	None	12/24 (50.0%)	12/24 (50.0%)	not estimable		⨁⨁ ◯◯ Go down ^a^	IMPORTANT
**Effects of interactive video game cycling on overweight and obese adolescent health**												
1	Randomized trials	Serious ^b^	It’s not serious	It’s not serious	It’s not serious	None	13/26 (50.0%)	13/26 (50.0%)	not estimable		⨁⨁⨁ ◯ Moderate ^b^	IMPORTANT
**Effects of an exercise intervention using Dance Revolution on endothelial function and other risk factors in overweight children**												
1	Randomized trials	Serious ^b^	It’s not serious	It’s not serious	It’s not serious	None	13/35 (37.1%)	22/35 (62.9%)	not estimable		⨁⨁⨁ ◯ Moderate ^b^	IMPORTANT
**Home-based exergaming among children with overweight and obesity: a randomized clinical trial**												
1	Randomized trials	Very serious ^a^	It’s not serious	It’s not serious	It’s not serious	None	23/46 (50.0%)	23/46 (50.0%)	not estimable		⨁⨁ ◯◯ Go down ^a^	IMPORTANT
**A randomized controlled trial of dance exergaming for exercise training in overweight and obese adolescent girls**												
1	Randomized trials	Serious ^b^	It’s not serious	It’s not serious	It’s not serious	None	22/41 (53.7%)	19/41 (46.3%)	not estimable		⨁⨁⨁ ◯ Moderate ^b^	NOT IMPORTANT
**Role of Exergame Play on Cardiorespiratory Fitness and Body Composition in Overweight and Obese Children**												
1	Randomized trials	Very serious ^a^	It’s not serious	It’s not serious	It’s not serious	None	21/31 (67.7%)	10/31 (32.3%)	not estimable		⨁⨁ ◯◯ Go down ^a^	IMPORTANT

^a^ High; ^b^ Some concerns ⨁⨁⨁⨁: Represents the quality of evidence on a 4-point scale. Each symbol corresponds to the following: ⨁⨁⨁⨁: High certainty; ⨁⨁⨁◯: Moderate certainty; ⨁⨁◯◯: Low certainty; ⨁◯◯◯: Very low certainty.

## Data Availability

The datasets generated during and/or analyzed during the current research are available from the Corresponding author upon reasonable request.

## Supplementary Materials

The following supporting information can be downloaded at: www.mdpi.com/article/10.3390/children12010029/s1. Supplementary Tables. Table S1. Studies report the effects of exergaming on morphological variables, biochemical variables, and blood pressure in children and adolescents with overweight/obesity. Supplementary Figures. Figure S1. Forest plot of changes in systolic blood pressure in children and adolescents participating in exergaming compared with children and adolescents assigned as controls. Values shown are effect sizes (Hedges’ g) with 95% confidence intervals (CI). The size of the squares plotted reflects the statistical weight of each study. Figure S2. Forest plot of changes in diastolic blood pressure in children and adolescents participating in exergaming compared with children and adolescents assigned as controls. Values shown are effect sizes (Hedges’ g) with 95% confidence intervals (CI). The size of the squares plotted reflects the statistical weight of each study. Figure S3. Forest plot of changes in HDL-cholesterol in children and adolescents participating in exergaming compared with children and adolescents assigned as controls. Values shown are effect sizes (Hedges’ g) with 95% confidence intervals (CI). The size of the squares plotted reflects the statistical weight of each study. Figure S4. Forest plot of changes in LDL-cholesterol in children and adolescents participating in exergaming compared with children and adolescents assigned as controls. Values shown are effect sizes (Hedges’ g) with 95% confidence intervals (CI). The size of the squares plotted reflects the statistical weight of each study. Figure S5. Forest plot of changes in total cholesterol in children and adolescents participating in exergaming compared with children and adolescents assigned as controls. Values shown are effect sizes (Hedges’ g) with 95% confidence intervals (CI). The size of the squares plotted reflects the statistical weight of each study. Figure S6. Forest plot of changes in triglycerides in children and adolescents participating in exergaming compared with children and adolescents assigned as controls. Values shown are effect sizes (Hedges’ g) with 95% confidence intervals (CI). The size of the squares plotted reflects the statistical weight of each study. Figure S7. Forest plot of changes in glucose in children and adolescents participating in exergaming compared with children and adolescents assigned as controls. Values shown are effect sizes (Hedges’ g) with 95% confidence intervals (CI). The size of the squares plotted reflects the statistical weight of each study. Figure S8. Forest plot of changes in BMI in children and adolescents participating in exergaming compared with children and adolescents assigned as controls. Values shown are effect sizes (Hedges’ g) with 95% confidence intervals (CI). The size of the squares plotted reflects the statistical weight of each study. Figure S9. Forest plot of changes in waist circumference in children and adolescents participating in exergaming compared with children and adolescents assigned as controls. Values shown are effect sizes (Hedges’ g) with 95% confidence intervals (CI). The size of the squares plotted reflects the statistical weight of each study. Figure S10. Forest plot of changes in body fat percentage in children and adolescents participating in exergaming compared with children and adolescents assigned as controls. Values shown are effect sizes (Hedges’ g) with 95% confidence intervals (CI). The size of the squares plotted reflects the statistical weight of each study.
