# Supplementary material for: Effects of Exergaming on Morphological Variables, Biochemical Parameters, and Blood Pressure in Children and Adolescents with Overweight/Obesity: A Systematic Review with Meta-Analysis of Randomized Controlled Trials
Source: Children (Basel). 2024 Dec 27;12(1):29. doi: 10.3390/children12010029 (PMC11763999; doi:10.3390/children12010029)
Supplement: Supplementary file 1 [file children-12-00029-s001.zip › children-3315990-supplementary figures.pdf]

Supplementary Figures

Study name	Hedges's g	Standard error	Variance	Lower limit	Upper limit	Z-Value	p-Value
	**Statistics for each study**						
Murphy et al. (2009)	0,100	0,348	0,121	-0,583	0,782	0,287	0,774
Staiano et al. (2017)	0,512	0,312	0,098	-0,100	1,124	1,638	0,101
Staiano et al. (2018)	0,797	0,302	0,091	0,206	1,388	2,643	0,008
Van biljon et al. (2021)	0,229	0,421	0,177	-0,596	1,054	0,544	0,586
	0,459	0,169	0,028	0,128	0,789	2,720	0,007

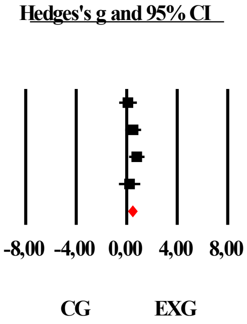

Figure S1. Forest plot of changes in systolic blood pressure in children and adolescents participating in exergaming compared with children and adolescents assigned as controls. Values shown are effect sizes (Hedges' g) with 95% confidence intervals (CI). The size of the squares plotted reflects the statistical weight of each study.

Study name	Hedges's g	Standard error	Variance	Lower limit	Upper limit	Z-Value	p-Value
	**Statistics for each study**						
Murphy et al. (2009)	-0,255	0,349	0,122	-0,939	0,430	-0,729	0,466
Staiano et al. (2017)	-0,305	0,309	0,095	-0,910	0,301	-0,987	0,324
Staiano et al. (2018)	1,153	0,314	0,098	0,538	1,768	3,676	0,000
Van biljon et al. (2021)	-0,324	0,422	0,178	-1,152	0,504	-0,768	0,442
	0,083	0,385	0,149	-0,673	0,838	0,215	0,830

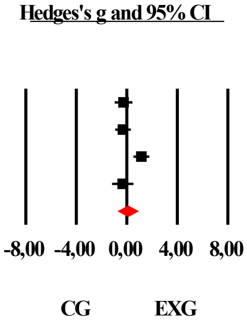

Figure S2. Forest plot of changes in diastolic blood pressure in children and adolescents participating in exergaming compared with children and adolescents assigned as controls. Values shown are effect sizes (Hedges' g) with 95% confidence intervals (CI). The size of the squares plotted reflects the statistical weight of each study.

Study name	Hedges's g	Standard error	Variance	Lower limit	Upper limit	Z-Value	p-Value
	Statistics for each study						
Abedelmalek et al. (2022)	-0,241	0,396	0,157	-1,016	0,535	-0,608	0,543
Adamo et al. (2010)	-0,908	0,476	0,227	-1,842	0,025	-1,907	0,057
Murphy et al. (2009)	-0,517	0,353	0,125	-1,210	0,175	-1,464	0,143
Staiano et al. (2017)	0,810	0,320	0,102	0,183	1,437	2,533	0,011
Staiano et al. (2018)	-0,356	0,292	0,085	-0,929	0,216	-1,219	0,223
	-0,206	0,292	0,085	-0,778	0,366	-0,705	0,481

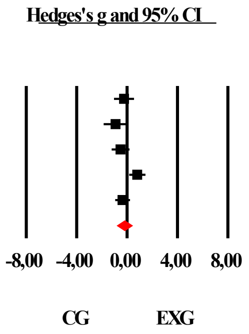

Figure S3. Forest plot of changes in HDL-cholesterol in children and adolescents participating in exergaming compared with children and adolescents assigned as controls. Values shown are effect sizes (Hedges' g) with 95% confidence intervals (CI). The size of the squares plotted reflects the statistical weight of each study.

Study name	Hedges's g	Standard error	Variance	Lower limit	Upper limit	Z-Value	p-Value
	Statistics for each study						
Abedelmalek et al. (2022)	0,261	0,396	0,157	-0,515	1,037	0,658	0,510
Adamo et al. (2010)	0,908	0,476	0,227	-0,025	1,842	1,907	0,057
Murphy et al. (2009)	0,232	0,349	0,122	-0,452	0,916	0,665	0,506
Staiano et al. (2017)	-0,542	0,313	0,098	-1,155	0,071	-1,732	0,083
Staiano et al. (2018)	0,833	0,303	0,092	0,240	1,426	2,753	0,006
	0,311	0,279	0,078	-0,235	0,857	1,115	0,265

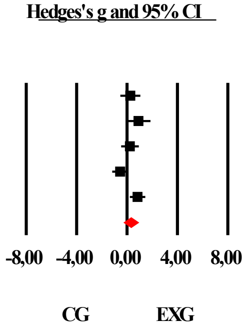

Figure S4. Forest plot of changes in LDL-cholesterol in children and adolescents participating in exergaming compared with children and adolescents assigned as controls. Values shown are effect sizes (Hedges' g) with 95% confidence intervals (CI). The size of the squares plotted reflects the statistical weight of each study.

Study name	Hedges's g	Standard error	Variance	Lower limit	Upper limit	Z-Value	p-Value
Abedelmalek et al. (2022)	0,892	0,415	0,172	0,079	1,705	2,151	0,031
Adamo et al. (2010)	0,451	0,458	0,210	-0,447	1,348	0,984	0,325
Murphy et al. (2009)	0,257	0,349	0,122	-0,427	0,942	0,736	0,462
Staiano et al. (2017)	-0,442	0,311	0,097	-1,051	0,168	-1,421	0,155
Staiano et al. (2018)	0,850	0,303	0,092	0,256	1,444	2,804	0,005
	0,382	0,267	0,072	-0,142	0,906	1,427	0,153

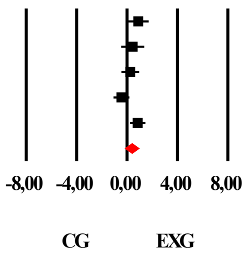

Figure S5. Forest plot of changes in total cholesterol in children and adolescents participating in exergaming compared with children and adolescents assigned as controls. Values shown are effect sizes (Hedges' g) with 95% confidence intervals (CI). The size of the squares plotted reflects the statistical weight of each study.

Study name	Hedges's g	Standard error	Variance	Lower limit	Upper limit	Z-Value	p-Value
Abedelmalek et al. (2022)	0,352	0,397	0,158	-0,427	1,131	0,885	0,376
Adamo et al. (2010)	0,587	0,462	0,214	-0,319	1,493	1,271	0,204
Murphy et al. (2009)	1,024	0,369	0,136	0,301	1,747	2,777	0,005
Staiano et al. (2017)	-0,594	0,314	0,099	-1,209	0,022	-1,891	0,059
Staiano et al. (2018)	0,799	0,302	0,091	0,208	1,390	2,649	0,008
	0,420	0,312	0,097	-0,190	1,031	1,350	0,177

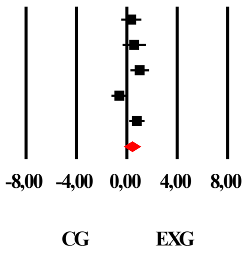

Figure S6. Forest plot of changes in triglycerides in children and adolescents participating in exergaming compared with children and adolescents assigned as controls. Values shown are effect sizes (Hedges' g) with 95% confidence intervals (CI). The size of the squares plotted reflects the statistical weight of each study.

Study name	Statistics for each study						
	Hedges's g	Standard error	Variance	Lower limit	Upper limit	Z-Value	p-Value
Murphy et al. (2009)	0,794	0,361	0,130	0,087	1,501	2,202	0,028
Staiano et al. (2017)	0,599	0,314	0,099	-0,017	1,215	1,907	0,057
Staiano et al. (2018)	-0,094	0,290	0,084	-0,662	0,475	-0,324	0,746
	0,406	0,277	0,077	-0,136	0,948	1,467	0,142

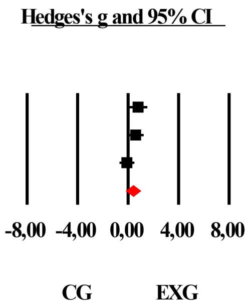

Figure S7. Forest plot of changes in glucose in children and adolescents participating in exergaming compared with children and adolescents assigned as controls. Values shown are effect sizes (Hedges' g) with 95% confidence intervals (CI). The size of the squares plotted reflects the statistical weight of each study.

Study name	Statistics for each study						
	Hedges's g	Standard error	Variance	Lower limit	Upper limit	Z-Value	p-Value
Adamo et al. (2010)	0,828	0,397	0,157	0,050	1,605	2,086	0,037
Staiano et al. (2017)	0,902	0,323	0,104	0,269	1,535	2,794	0,005
Staiano et al. (2018)	0,526	0,295	0,087	-0,052	1,104	1,784	0,074
Van biljon et al. (2021)	-0,357	0,423	0,179	-1,186	0,473	-0,843	0,399
	0,512	0,256	0,066	0,010	1,015	1,999	0,046

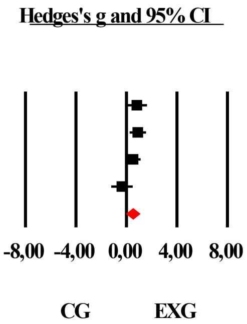

Figure S8. Forest plot of changes in BMI in children and adolescents participating in exergaming compared with children and adolescents assigned as controls. Values shown are effect sizes (Hedges' g) with 95% confidence intervals (CI). The size of the squares plotted reflects the statistical weight of each study.

Study name	Hedges's g	Standard error	Variance	Lower limit	Upper limit	Z-Value	p-Value
Abedelmalek et al. (2022)	-0,012	0,394	0,155	-0,784	0,761	-0,030	0,976
Adamo et al. (2010)	1,016	0,405	0,164	0,222	1,810	2,508	0,012
Staiano et al. (2017)	0,902	0,323	0,104	0,269	1,535	2,794	0,005
	0,649	0,313	0,098	0,035	1,263	2,072	0,038

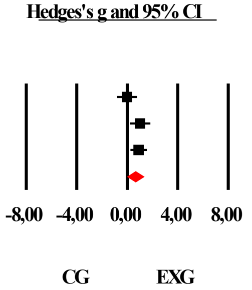

Figure S9. Forest plot of changes in waist circumference in children and adolescents participating in exergaming compared with children and adolescents assigned as controls. Values shown are effect sizes (Hedges' g) with 95% confidence intervals (CI). The size of the squares plotted reflects the statistical weight of each study.

Study name	Hedges's g	Standard error	Variance	Lower limit	Upper limit	Z-Value	p-Value
Abedelmalek et al. (2022)	0,257	0,396	0,157	-0,519	1,033	0,648	0,517
Adamo et al. (2010)	0,037	0,380	0,144	-0,707	0,782	0,099	0,921
Staiano et al. (2017)	-0,174	0,308	0,095	-0,777	0,429	-0,565	0,572
	0,003	0,205	0,042	-0,399	0,404	0,013	0,990

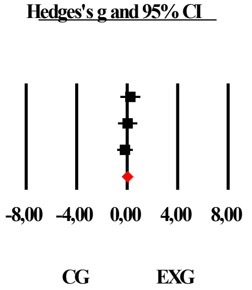

Figure S10. Forest plot of changes in body fat percentage in children and adolescents participating in exergaming compared with children and adolescents assigned as controls. Values shown are effect sizes (Hedges' g) with 95% confidence intervals (CI). The size of the squares plotted reflects the statistical weight of each study.

Analysis by subgroups

By age

In <15 years of age there are significant differences ($p = 0.006$) in favor of EXG in total cholesterol (3 experimental groups; ES = 0.56; 95% CI = 0.16 to 0.96; within group $I^2 = 0.00\%$), LDL-cholesterol ($p = 0.01$) (3 experimental groups; ES = 0.63; 95% CI = 0. 22 to 1.05; within group $I^2 = 4.33\%$), HDL-cholesterol ($p = 0.01$) (3 experimental groups; ES = 0.51; 95% CI = -0.41 to 0.11; within group $I^2 = 0.00\%$), triglycerides ($p = 0.000$) (3 experimental groups; ES = 0.82; 95% CI = 0.41 to 1.23 within group $I^2 = 0.00\%$).

In those ≥15 years of age, there were no significant differences in total cholesterol (3 experimental groups; ES = 0.19; 95% CI = -1.10 to 1.50; within group I^2 = 84.8%), LDL-cholesterol (p = 0.65) (3 experimental groups; ES = -0.17; 95% CI = -0. 96 to 0.60; within group I^2 = 60.4%), HDL-cholesterol (p = 0.55) (3 experimental groups; ES = -0.31; 95% CI = -0.71 to 1.33; within group I^2 = 76.5%), triglycerides (p = 0.74) (3 experimental groups; ES = -0.15; 95% CI = -1.07 to 0.77 within group I^2 = 71.3%).

Duration of training

In <12 weeks only significant differences in favor of EXG are presented in total cholesterol (p = 0.02) (3 experimental groups; ES = 0.69; 95% CI = 0.09 to 0.91; within-group I^2 = 0.00%), with no significant differences in LDL-cholesterol (p = 0.09) (3 experimental groups; ES = 0. 53; 95% CI = -0.09 to 1.15; within-group I^2 = 8.53%), HDL-cholesterol (p = 0.11) (3 experimental groups; ES = -0.52; 95% CI = -1.16 to 0.12; within-group I^2 = 13.9%), triglycerides (p = 0.13) (3 experimental groups; ES = 0.45; 95% CI = -0.13 to 1.04 within-group I^2 = 0.00%).

At ≥12 weeks, no significant differences in favor of EXG in total cholesterol (p = 0.56) (3 experimental groups; ES = 0.22; 95% CI = -0.54 to 0.98; within-group I^2 = 77.4%), LDL cholesterol (p = 0.67) (3 experimental groups; ES = 0.17; 95% CI = -0.63 to 0.98; within-group I^2 = 80%), HDL cholesterol (p = 0.96) (3 experimental groups; ES = -0.01; 95% CI = -0.83 to 0.79; within group I^2 = 80%), triglycerides (p = 0.43) (3 experimental groups; ES = 0.40; 95% CI = -0.59 to 1.40 within group I^2 = 86.2%).

Frequency of training

At <3 sessions per week, there are no significant differences in favor of EXG in total cholesterol (p = 0.51) (3 experimental groups; ES = 0.27; 95% CI = -0.56 to 1.12; within group I^2 = 77.9%), LDL-cholesterol (p = 0.45) (3 experimental groups; ES = 0. 37; 95% CI = -0.61 to 1.35; within group I^2 = 83.2%), HDL-cholesterol (p = 0.81) (3 experimental groups; ES = -0.11; 95% CI = -1.07 to 0.84; within group I^2 = 82.6%), triglycerides (p = 0.60) (3 experimental groups; ES = 0.24; 95% CI = -0.68 to 1.18 within group I^2 = 81.8%).

In ≥3 sessions per week, only significant differences in favor of EXG are presented in triglycerides (p = 0.03) (3 experimental groups; ES = 0.70; 95% CI = -0.04 to 1.36; within group I^2 = 35%), with no significant differences in total cholesterol (p = 0.08) (3 experimental groups; ES = 0. 53; 95% CI = --0.08 to 1.15; within group I^2 = 27%), LDL-cholesterol (p = 0.35) (3 experimental groups; ES = 0.24; 95% CI = -0.26 to 0.75; within group I^2 = 0.00%), HDL-cholesterol (p = 0.13) (3 experimental groups; ES = -0.39; 95% CI = -0.91 to 0.12 within group I^2 = 0.00%).
