# Supplementary material for: Effects of Exergaming on Morphological Variables, Biochemical Parameters, and Blood Pressure in Children and Adolescents with Overweight/Obesity: A Systematic Review with Meta-Analysis of Randomized Controlled Trials
Source: Children (Basel). 2024 Dec 27;12(1):29. doi: 10.3390/children12010029 (PMC11763999; doi:10.3390/children12010029)
Supplement: Supplementary file 1 [file children-12-00029-s001.zip › children-3315990-supplementary tables.pdf]

Supplementary Tables

Table S1. Studies report the effects of exergaming on morphological variables, biochemical variables, and blood pressure in children and adolescents with overweight/obesity.

Study	Country	Study design	Sample's initial health	Groups (n and % female or male)	Mean age (years)	Weight (kg)/Heigh (m)	Type of interventions and control groups	Training Volume			Training Intensity	Morphological variables	Biochemical parameters	Blood pressure
								Weeks	Frequency (sessions/weeks)	Session duration (minutes)				
Abedelmalek, *et al.* [35]	Tunisia	RCT	Adolescents with obesity	EXG: 12<br>Active CG: 12<br>NR sex	EXG: 15.75 ± 0.8<br>CG: 16.25 ± 0.8	98.5± 1.1/ 1.67±0.2<br>99.4± 1.4/1.65±0.3	EXG: Cooperative EXG (sports games) while following intermittent fasting.<br>CG: fasting rituals without additional exercise	4	5	45	50% to 70% of maximum heart rate	-Body weight (kg)<br>- BMI (kg·m−2)<br>-BFP<br>-Waist circumference (cm)<br>-Fat mass (g/d)	-Total cholesterol (mmol. l−1)<br>-LDL (mmol. l−1)<br>-HDL (mmol. l−1)<br>-Triglycerides (mmol. l−1)	-SBP (mmHg)<br>-DBP (mmHg)
Staiano, *et al.* [14]	United States	RCT	Children with overweight/obesity	EXG:23<br>Inactive CG:23<br>NR sex	Both groups 11.2 ± 0.8	NR/NR	EXG: Active EXG with telecoaching (Xbox Kinect and Xbox 360).<br>CG: no intervention was continued with her daily activities.	24	3	60	heart rate monitoring (accelerometers).	-Body weight (z-score)<br>-Body mass index (z-score)<br>-BFP	-Total cholesterol mg/dL<br>-HDL mg/dL<br>-LDL mg/dL<br>Triglycerides mg/dL<br>- Glucose mg/dL	NR
Staiano, *et al.* [33]	United States	RCT	Overweight/obese girls	EXG: 22<br>Inactive CG:19<br>NR sex	EXG: 15.3 ± 1.2<br>CG: 16.1 ± 1.4	97.2±27.3/1.60±5.9<br>101.1±26.1/164±5.8	EXG: structured exergaming sessions based on group dance. (Xbox Kinect and Xbox 360).<br>CG: no intervention was continued with her daily activities.	12	3	60	heart rate monitoring (between 60 and 75% of maximum heart rate)	-Body Mass Index (z-score)<br>-Waist Circumference (cm)<br>-BFP<br>-Total Body Weight (kg)	-Total cholesterol (mg dL−1)<br>-HDL (mg dL−1)<br>-LDL (mg dL−1)<br>-Triglycerides (mg dL−1)<br>-Glucose (mg dL−1)<br>-Insulin (mg dL−1)	-SBP (mmHg)<br>-DBP (mmHg)
van Biljon, *et al.* [34]	South Africa	RCT	Obese Children	EXG:11<br>Inactive CG:10<br>NR sex	Both groups 11.40±0.86	NR/NR	EXG: exergaming sessions with the Nintendo Wii™ console, using boxing and hula hoop games.<br>CG: no intervention was continued with her daily activities.	6	3	30	NR	-Body mass index (percentile)	-SBP (mmHg)<br>-DBP (mmHg)	-SBP (mmHg)<br>-DBP (mmHg)

Adamo, *et al.* [36]	Canada	RCT	Adolescents with overweight/obesity	EXG:13<br><br>Active CG: 13<br><br>53% female<br>47% male	EXG: 13.9±1.4<br><br>CG: 15.1±1.8	93.1±20.8/ 1.63±4.1<br><br>105.3±23.8/1.65±8.0	EXG: a stationary bicycle connected to a video game (PlayStation 2).<br><br>CG: bicycle, but without the video game.	10	2	60	Heart rate monitoring.	-Body Weight (kg)<br><br>-Body mass index (kg m–2)<br>-Waist circumference (cm)<br>-Fat mass (kg)<br>-BFP<br>-Fat-free mass (kg)	-Total cholesterol (mmol L–1)<br>-HDL (mmol L–1)<br>-LDL (mmol L–1)<br>-Glucose (mmol L–1)<br>-Insulin (mmol L–1)<br>-Triglycerides (mmol L–1)	NR
Murphy, *et al.* [37]	United States	RCT	Overweight children	EXG:23<br><br>Inactive CG:12<br><br>48% female<br>52% male	Both groups 10.21 ± 1.67	62.5±15.3/1.48±10.4<br><br>69.5±17.0/ 147±7.5	EXG: Exercises are done through the video game "Dance Dance Revolution" (rhythmic physical movements). (PlayStation 2).<br><br>CG: no intervention was continued with her daily activities.	12	3 to 5	30	CERT	-Body mass index (kg m–2)<br><br>-Body weight (kg)	-Total cholesterol (mg/dl)<br>-LDL (mg/dl)<br><br>-HDL (mg/dl)<br><br>-Triglycerides (mg/dl)<br><br>-Glucose (mg/dl)<br><br>-Insulin (mg/dl)	-SBP (mmHg)<br>-DBP (mmHg)

EXG: exergaming; CG: control group; NR: no reported; RCT: randomized controlled trial; BFP: body fat percentage; LDL: low-density lipoprotein; HDL: high-density lipoprotein; SBP: systolic blood pressure; DBP: diastolic blood pressure; CERT: Child's effort rating table.
